# Prenatal Cannabis Use and Offspring Autism-Related Behaviors: Examining Maternal Stress as a Moderator in a Black American Cohort

**DOI:** 10.1007/s10803-023-05982-z

**Published:** 2023-04-25

**Authors:** C. Nutor, A. Dunlop, O. Sadler, P. A. Brennan

**Affiliations:** 1 Department of Psychology, Emory University, 36 Eagle Row, Atlanta, GA 30322 USA; 2 Department of Gynecology and Obstetrics, Emory University, 1365 E Clifton Rd NE, Atlanta, GA 30322 USA

**Keywords:** Autism spectrum disorder, Prenatal cannabis use, Prenatal stress, Minority mental health, Neurodevelopment

## Abstract

Prenatal cannabis use and maternal stress have been proposed as risk factors for autism spectrum disorder (ASD). Black mothers and mothers of lower socioeconomic status (SES) may be especially likely to experience high levels of stress. This study examined the impact of prenatal cannabis use and maternal stress (i.e., prenatal distress, racial discrimination, and lower SES) on child ASD-related behaviors in a sample of 172 Black mother-child pairs. We found that prenatal stress was significantly associated with ASD-related behaviors. Prenatal cannabis use did not predict ASD-related behaviors and did not interact with maternal stress to predict ASD-related behaviors. These findings replicate previous work on prenatal stress-ASD associations and add to the limited literature on prenatal cannabis-ASD associations in Black samples.

**Supplementary Information:**

The online version contains supplementary material available at 10.1007/s10803-023-05982-z.

According to the Diagnostic Statistical Manual, Fifth Edition (DSM-5), autism spectrum disorder (ASD) represents a wide continuum of associated social and neurobehavioral deficits, including social communication deficits with restricted and repetitive patterns of behavior (American Psychiatric Association, 2013). The reported prevalence of ASD has increased in the U.S. in the last several decades, with recent estimates as high as 1 in 54 children at age 8 (Maenner et al., 2020). Of note, ASD prevalence rates have historically been lower for Black children and children with parents of low socioeconomic status (SES). For example, population-based surveillance data from 11 sites across the U.S. in 2012 indicate that the estimated prevalence of ASD in non-Hispanic white children was 20% higher than non-Hispanic Black children (Imm et al., 2019). Additionally, ASD prevalence was lowest in the low parental education attainment tertile and highest in the high parental education attainment tertile, consistent with a dose–response association between census tract educational attainment and population-based ASD prevalence in 2010 (Durkin et al., 2017). Importantly, these associations are specific to diagnoses of ASD, which may require treatment seeking and access to care—resources which might be limited Black and low SES populations due to systemic barriers (Broder-Fingert et al., 2020).

The field of psychopathology is largely moving towards a more dimensional conceptualization rather than dichotomous disorder diagnoses (Insel et al., 2010). With the shift to this new conceptualization, researchers have found that racial minority parents and parents with lower educational attainment report increased observations of ASD behaviors in their children compared to their White and more educated counterparts, despite the lower prevalence of ASD diagnoses noted above (Khowaja et al., 2015). This contrast may reflect higher rates of misdiagnosis among Black children (Mandell et al., 2007) and higher levels of health care access and service delivery in non-Hispanic white and higher SES communities (Bishop-Fitzpatrick & Kind, 2017; Chiang et al., 2018; Khowaja et al., 2015). For example, Constantino et al. (2020) found that Black American parents reported developmental concerns about their child an average of three years before an ASD diagnosis was made. As the field has moved toward examining psychopathology on a continuum, it is increasingly important to examine potential predictors of ASD-related *behaviors* rather than *diagnoses*.

Importantly, the DSM-5 specifies that ASD symptoms emerge in the early developmental period of childhood (American Psychiatric Association, 2013). Although twin and family studies suggest a strong genetic component in the etiology of ASD, recent evidence indicates that up to 40–50% of variance in ASD may be determined by environmental factors (Modabbernia et al., 2017). A significant role for environmental factors in the etiology of ASD is also consistent with the rapidly increasing rates of this disorder over the past few decades (Hertz-Picciotto et al., 2018). The Developmental Origins of Health and Disease hypothesis suggests that environmental influences on psychopathology may originate in the prenatal period, underlining the importance of investigating mechanisms of fetal exposure and other perinatal factors in early neurodevelopment (Barker, 2007). In line with this theory, increased attention has focused on the role that prenatal exposures may play in increasing the risk of ASD. The current study will focus on two of these potential prenatal environmental influences: maternal cannabis use and stress.

With the legalization of cannabis in various states in the U.S., there has been a decrease in the perception of risk and a concomitant increase in reports of cannabis use by pregnant women (Jarlenski et al., 2017; Westfall et al., 2006). For example, Brown et al. (2017) found that prevalence of cannabis use among pregnant women increased 62% from 2002 to 2014. There are also reports that prenatal cannabis use may have increased with the COVID-19 pandemic (Young-Wolff et al., 2021). Importantly, cannabis potency has also increased in recent years with some confiscated samples containing more than 80% Tetrahydrocannabinol (THC), the psychoactive component of cannabis (Mehmedic et al., 2010). Results from pre-clinical, non-human animal studies indicate that THC can pass the placental barrier and reach the fetus; and fetal exposure to cannabis in humans has been linked to poorer neonatal and cognitive outcomes such as preterm birth, low birth weight, hyperactivity, and poorer attention (Frau et al., 2019; Nashed et al., 2021). In light of these findings and in conjunction with the reported increased potency and increased prenatal use, it is imperative to better understand how prenatal cannabis exposure may influence child neurodevelopment.

Only a few empirical studies have tested the association between prenatal cannabis use and ASD. In 2015, Wood et al. found that first trimester cannabis use predicted higher offspring ASD scores using a behavioral scale in a small sample of children co-exposed to anticonvulsant medications. Because the main goal of this study was to evaluate the association between ASD and prenatal anticonvulsant exposure, the authors were unable to examine the effect of cannabis without co-exposure. In 2020, Corsi et al. found that children of mothers who retrospectively reported prenatal cannabis use were 50% more likely to receive an ASD diagnosis than children with no reported prenatal exposure to cannabis. The inclusion of relevant covariates (including other substance use, preterm birth, and income) dampened these effects, although findings held. More recently, DiGuiseppi et al. (2021) found that maternal self-reported use of cannabis in the perinatal period was not associated with ASD, after accounting for maternal education and peri-pregnancy tobacco and alcohol use. Authors called for further research to replicate these studies using a prospective design with controls for potential confounds—a gap the current study fills.

The association between maternal prenatal distress and offspring symptoms of ASD has been demonstrated across different species, research designs, and types of prenatal distress (Kinney et al., 2008). In particular, stress-related factors such as maternal depression and anxiety have been associated with increased risk for ASD (Amiri et al., 2020; Chen et al., 2020; Hagberg et al., 2018). Studies also show that stress-related factors such as maternal anxiety, depression, and trauma are associated with higher odds of cannabis use among pregnant women. A recent study suggested a dose-response association, with higher odds of cannabis use associated with co-occurring depressive and anxiety disorders and greater depression severity (Young-Wolff et al., 2020). Because maternal distress during pregnancy is associated with both higher odds of ASD and higher odds of prenatal cannabis use, we propose that prenatal distress will moderate the association between prenatal cannabis use and ASD behaviors.

While a variety of maternal stressors have been examined in the context of ASD, studies have largely overlooked the potential impact of maternal race-related stress. A recent study that directly compared stressors experienced by non-Hispanic White and Black women in pregnancy found that non-Hispanic Black women experienced higher levels of biological indicators of stress, relative to non-Hispanic White women of the same level of SES (Borders et al., 2015). The current study tests the novel hypothesis that maternal reports of perceived racial discrimination will associate with child ASD behaviors, both independently and in combination with prenatal cannabis exposure.

Epidemiological studies in the United States have historically found that ASD is more prevalent in high SES families, but more recently, a study in California found that this trend of higher rates of ASD in higher SES communities reversed between 1992 and 2016 such that low SES individuals were more likely to receive ASD diagnoses (Winter et al., 2020). Socioeconomic status is also associated with higher levels of psychological stress, especially for Black people (Cundiff et al., 2022). Because these children are already at a disadvantage in under-resourced conditions, it is reasonable to hypothesize that the negative impacts of prenatal cannabis exposures would be more prominent in children from low SES backgrounds. For instance, Khoury et al. (2018), found that low and mixed prenatal SES were associated with increased risk of externalizing behavior problems as a result of prenatal alcohol exposure compared to those from mid-SES families. Therefore, evidence supports that cannabis-exposed infants may develop differently depending on their family’s level of SES.

We hypothesize that:

1. Prenatal cannabis use will predict increased levels of child ASD-related behaviors, and this association will remain significant when covariates such as child biological sex, socioeconomic status, and maternal tobacco and alcohol use in pregnancy are controlled.
2. Maternal stress—conceptualized as maternal prenatal distress, racial discrimination, and low SES—will predict increased levels of child ASD-related behaviors and this association will remain significant when covariates such as child biological sex, and maternal tobacco and alcohol use in pregnancy are controlled.
3. Maternal stress will moderate the association between prenatal cannabis use and child ASD-related behaviors. We hypothesize that positive associations between prenatal cannabis use and child ASD-related behaviors will be stronger in cases of (a) higher maternal prenatal distress, (b) higher racial discrimination, and (c) lower SES.

## Methods

### Participants

The participants in this study were 172 Black American mothers and their children. Participants were recruited from an ongoing longitudinal study that follows Black American women through pregnancy (Corwin et al., 2017) and again at child ages two through six years (Padula et al., 2020). Women were recruited for the larger ongoing prenatal study during the first trimester of pregnancy from Grady Memorial Hospital, a public hospital, and Emory Midtown Hospital, a private hospital in Atlanta, resulting in a socioeconomically diverse sample. Exclusion criteria for follow-up were: (1) non-singleton birth, and (2) major congenital anomalies.

### Procedures

Pregnant Black women (ages 18–40 years) were recruited from the Emory and Grady prenatal clinics at 8–14 weeks’ gestation (as determined by standard criteria based upon last menstrual period and/or first trimester ultrasound). In the context of the prenatal study, mothers reported on their cannabis use and stress at two timepoints in pregnancy: between 8 and 14 weeks and between 24 and 30 weeks. Medical records were abstracted following the birth of the child, and included maternal reports of cannabis use to clinical providers during prenatal care. Mothers were then invited to participate in a separate study on child health outcomes when their children reached the age of two years. ASD behaviors were assessed in the child follow up study as described in detail below.

### Measures

#### Maternal Prenatal Substance Exposure

Pregnant women reported substance use during pregnancy using the following measures:

A questionnaire based on the *Timeline Follow-back Interview (*TLFB) was administered to the mothers twice during pregnancy to assess prenatal substance use. This survey assesses the use (yes/no) of a variety of drugs (i.e., tobacco, cannabis, alcohol, cocaine, etc.) during the last month. (Sobell & Sobell, 1992).

*Medical Record Review* Maternal reports of cannabis use were also obtained from clinical prenatal care provider notes within the medical record. Providers conduct a ‘clinical interview’ during the initial prenatal appointment where they gathered an in-depth medical history. Provider documentation typically indicates “positive for marijuana use,” “uses marijuana,” or “no substance use”. Providers had the ability to update the history and document substance use that occurred after the initial prenatal care encounter.

In the current study, we used categorical measures of cannabis, tobacco, alcohol, and other drug use (yes/no) across pregnancy.

We collapsed substance use reported via self-report questionnaires and medical record review such that substance use on either measure was coded as yes. In our sample, 35% of mothers were categorized as using cannabis, 18% as using tobacco, 10% as drinking alcohol, and 1% as using other illicit substances.

#### Maternal Prenatal Distress

Pregnant women provided self-report measures of perceived stress, anxiety, and depressive symptoms at two prenatal visits (in mid and late pregnancy) using the following measures:

*Perceived Stress Scale* (PSS) is a 14-item questionnaire that measures experiences of stress in the last month. PSS scores significantly correlate with HPA axis function, a biological proxy for stress, during pregnancy and postpartum (Cohen et al., 1983).

*Spielberger State Trait Anxiety Inventory* (STAI) measures current stress and anxiety as well as anger traits using a 40-item inventory (scored 0–1). It has been widely used in perinatal studies and is well-validated in minority and low-literacy populations stress (Gaudry et al., 1975; Spielberger et al., 1970). In the current study, we administered the 20 state anxiety items only, to capture prenatal anxiety symptoms.

*Edinburgh Depression Scale* (EDS) is a 10-item scale that assesses depressive symptoms. This measure has been shown to have acceptable sensitivity and specificity in community samples and good construct validity when compared with structured clinical interviews (Cox et al., 1987).

Principal components analyses (PCA) were conducted to assess whether psychosocial stress, early life stress, and socioeconomic measures loaded onto distinct factors, and in order to consolidate measures to reduce multiple testing and Type I error. The PCA revealed that the PSS, STAI and EDS measures loaded together onto a single factor as described in Hendrix et al. (2021) and detailed in Supplemental Analyses. Therefore, we used the factor score from the PCA for our prenatal maternal distress variable.

#### Socioeconomic Status

SES was measured via maternal self-reports of education, income, marital status, and insurance type. A factor score based on these measures was derived as the second component of the PCA described above (see Supplemental Analyses)—this factor score was used in as the measure of SES in the current study.

#### Racial Discrimination

Pregnant women self-reported their experiences of discrimination at the first prenatal visit on two measures as follows:

*Jackson, Hogue, Phillips Contextualized Stress Measure* (JHP) is a measure designed to assess chronic intersectional racial and gendered stress (Jackson, 2005). This tool is a multidimensional measure created from focus groups and interviews as part of community-based participatory research where Black American women were asked to elaborate on their particular racial and gendered stressors and stress mediators. The JHP consists of subscales for assessing racism, gendered roles and burden, abuse and neglect, workplace stress, coping, social support, and affective stress responses (distress). The racism subscale, used in this study, contains five items rated on a likert scale reflecting how much the respondent agrees with statements such as: “I have to work harder than white women to earn equal recognition” and “Racism is a problem in my life.”

The *Krieger Experiences of Discrimination Scale* assesses self-reported experiences of race-based discrimination across the lifespan (Krieger et al., 2005). On this measure, participants were asked whether, and how many times, they had been discriminated against in 9 different situations (e.g., “getting hired or getting a job” and “getting services in a store or restaurant”). Consistent with epidemiological research showing that experiencing discrimination across a greater number of different situations predicts psychological symptoms among AA women (Ertel et al., 2012). We used the discrimination summary score, which reflects the number of different situations in which women reported that they had experienced discrimination. Scores ranged from 0 to 9, with higher scores representing discrimination in more situations.

In our sample, discrimination scores were correlated with each other (r=. 33); to reduce repeated testing related to this component of maternal stress, we used a combined racism score calculated by summing the standardized scores from the JHP racism scale and the Krieger summary score.

#### Autism Spectrum Disorder (ASD) Behaviors

At the age 2 follow-up, children were assessed for ASD-related behaviors using three separate measures as follows:

*Autism Diagnostic Observation Schedule* (ADOS) is a semi-structured, clinician-administered assessment of communication, social interaction, and play (Lord et al., 2000). Using developmentally appropriate modules, children were asked to do a number of tasks, such as tell a story or play a make-believe game. Each task loads onto one of three domains: Communication (e.g., conversation, reporting of events), Reciprocal Social Interaction (e.g., quality of social response, overall quality of rapport), and Stereotyped Behaviors and Restricted Interests (e.g., Unusual sensory interest in play material/person). A total ADOS score provides a continuous measure of DSM-5 ASD symptoms and was used for this study.

The *Modified Checklist for Autism in Toddlers* (M-CHAT) was also electronically administered to the mothers alongside other developmental surveys at the 2 year visit. The M-CHAT is a 20-item autism screener that assesses behaviors indicative of ASD. Example items include: “If you point at something across the room, does your child look at it?” and “Does your child play pretend or make-believe?” (Robins et al., 2001). Total scores on the M-CHAT were used as a continuous measure of ASD behaviors in this study.

The *Achenbach Child Behavior Checklist* (CBCL) *1.5-5* was completed by the mothers at the age 2 follow up, focusing on her observations of her child’s behavior. The CBCL is a widely used measure for assessing child behavior and has been shown to have excellent reliability and validity (Achenbach & Rescorla, 2000). For this study we used the continuous raw score on the Pervasive Developmental Problems (PDP) scale, which was constructed based on DSM-IV criteria and consists of 13 items (Cronbach’s alpha = 0.75; Achenbach, Dumenci, & Rescorla, 2003). Example items of the PDP scale include: “avoids looking others in the eye” and “afraid to try new things.” Mothers rated items as 0 = not true, 1 = somewhat or sometimes true, or 2 = very true or often true based on the previous 2 months. The PDP scale has been conceptualized as an ASD screener, and studies demonstrate that children with ASD score significantly higher on the PDP scale compared to both low- and high-risk counterparts (Rescorla et al., 2019).

Pearson correlations indicated that ASD measures used in this study were significantly correlated with each other, but the magnitude of the correlations was small (r = .16 between ADOS and CBCL; r = .21 for correlation between M-CHAT and ADOS; r = .31 for correlation between M-CHAT and CBCL). These lower correlations were not surprising given that the measures had distinct raters, and some were screeners for ASD rather than diagnostic measures of ASD. We therefore opted to evaluate each ASD behavior measure separately rather than applying a latent variable approach.

### Data Analysis

Participants were included in the final dataset if they had prenatal cannabis use data and at least one measure of ASD-related behaviors. 172 participants met this criteria (see Supplementary Material). In early pregnancy, *n =* 163 participants completed the JHP discrimination measure. At age 2, all but one participant (*n* = 171) completed the CBCL, *n* = 164 for the ADOS, and *n* = 126 for the M-CHAT data. Participants who did not have available ASD data were older (M_age_ = 26.50, SD = 5 0.26) than participants with these data (M_age_ = 24.72, SD = 4.30, *t* = 2.09, p = .04). There were no other significant differences between included and excluded participants (all *p*’s > 0.05). We concluded that our data met Missing At Random (MAR) specifications (Nicholson et al., 2017). Hereafter, all results reported in the text, Tables, and Figures are based on multiply imputed data.

We included child sex, tobacco, alcohol, and other drug exposures during pregnancy in all models. These covariates were determined a priori based on previous literature. For the first hypothesis, we also included maternal SES in the models given the significant correlations between maternal education and marital status (proxies for SES) and cannabis use.

We conducted analyses of covariance (ANCOVAs) to test if prenatal cannabis use, a categorical variable, would predict ASD-related behaviors in children that were measured continuously. Because maternal prenatal distress was also measured continuously, we conducted linear regressions to test if maternal stress predicts ASD-related behaviors. Using SPSS PROCESS, we examined whether interactions between prenatal cannabis use and each stressor significantly predicted ASD-related behaviors above and beyond main effects. Each maternal stress variable was examined in a separate regression model. Statistical significance was determined by a threshold of p < .05.

## Results

### Descriptive Statistics and Exploratory Correlations

Table 1 displays descriptive statistics for the study variables in the sample. In this table, we compared cannabis users to non-cannabis users. Of note, there was a statistically significant difference in preterm birth, marital status, education level, and prenatal tobacco use between mothers who used cannabis and those who did not. These differences were expected based on the literature. No other group differences were significant.

**Table 1:** Descriptive charactristics of sample divided by prenatal cannabis use

Total sample N = 172	Cannabis users N = 60	Non-cannabis users N = 112	Significance (p-values)
Sex, N (%)			
Male	32 (53)	56 (50)	.68
Preterm birth N (%)			
Yes	13 (22)	11 (10)	.04*
Gestational age at birth, M (SD)	38.40 (2.47)	38.70 (1.65)	.35
Birthweight (kg), M (SD)	2991.22 (567.2)	3134.10 (491.38)	.09
Maternal age, M (SD)	24.60 (4.67)	25.54 (4.61)	.20
Marital status, N (%)			
Single	58 (97)	89 (80)	.002**
Education, N (%)			
High school diploma or less	43 (72)	48 (43)	< .001**
Income, N (%)			
Below the poverty level	32 (53)	45 (40)	.10
Insurance type, N (%)			
Medicaid	27 (45)	40 (36)	.23
Prenatal tobacco use, N (%)	22 (37)	9 (8)	< .001**
Prenatal alcohol use, N (%)	8 (13)	9 (8)	.27
Prenatal other drug use, N (%)	1 (2)	0 (0)	.17
Experiences of discrimination, M (SD)	2.03 (2.44)	1.97 (2.19)	.87
Racial and gendered stress, M (SD)	13.70 (4.68)	13.68 (4.45)	.98

*Correlation is significant at the 0.05 level (2-tailed)

**Correlation is significant at the 0.01 level (2-tailed)

Table 2 displays bivariate correlations between the ASD measures, the covariates, and proposed moderators. The ADOS was significantly correlated with child sex and tobacco use during pregnancy. We did not observe these expected associations with the other maternal report measures of ASD-related behaviors (M-CHAT and CBCL).

**Table 2:** Bivariate correlations between covariates, moderators, and autism measures

		Sex	Cannabis	Alcohol	Tobacco	Other drugs	Maternal stress	SES	Racism
ADOS	Pearson Correlation	− .22	.02	.07	.16	.13	− .03	− .11	− .05
	Sig. (2-tailed)	.003**	.75	.37	.04*	.09	.67	.16	.48
CBCL	Pearson Correlation	−.10	.13	− .11	.06	.08	.27	− .14	.05
	Sig. (2-tailed)	.18	.08	.17	.47	.30	< .001**	.07	.55
MCHAT	Pearson Correlation	− .09	− .03	.02	.08	.06	.08	− .08	− .04
	Sig. (2-tailed)	.24	.67	.80	.28	.43	.25	.33	.59

*Correlation is significant at the 0.05 level (2-tailed)

**Correlation is significant at the 0.01 level (2-tailed)

#### Prenatal Cannabis Use and ASD-Related Behaviors

With covariates included, prenatal cannabis did not predict the ASD-related behaviors measured by the ADOS (F_(1,165)_ = 1.50, p = .22), M-CHAT (F_(1,165)_ = 2.77, p = .10), or CBCL (F_(1,165)_ = 1.50, p = .22; Table 3). Across measures, there was no evidence that self-reported prenatal cannabis exposure affects child ASD-related behaviors. Results were similar when marital status, insurance, income, and education were entered as separate covariates.

**Table 3:** ANCOVA between prenatal cannabis use and autism behaviors

	ADOS			M-CHAT			CBCL		
	Partial η^2^	F Statistic	Sig	Partial η^2^	F Statistic	Sig	Partial η^2^	F Statistic	Sig
*Block 1: Covariates*									
Biological sex	.06	10.5	.001**	.01	1.74	.19	.01	1.31	.26
SES	.01	1.33	.25	.007	1.23	.27	.01	1.19	.28
Prenatal tobacco use	.03	4.17	.04*	.01	1.78	.18	.002	.25	.62
Prenatal alcohol use	< .001	.002	.96	< .001	.01	.94	.02	2.72	.10
Prenatal other drug use	.02	2.56	.11	.003	.49	.49	.01	1.31	.25
*Block 2: Main effect*									
Prenatal cannabis use	.01	1.50	.22	.02	2.77	.10	.01	1.50	.22

*Correlation is significant at the 0.05 level (2-tailed)

**Correlation is significant at the 0.01 level (2-tailed)

#### Maternal Stress and ASD-Related Behaviors

We found that prenatal maternal distress predicted ASD-related behaviors reported on the CBCL (DR^2^ = 0.07, t = 3.64, p < .001), but not the ADOS (DR^2^ = 0.001, t = − 0.33, p = .74) or the M-CHAT (DR^2^ = 0.01, t = 0.89, p = .37; Table 4). Experiences of racial discrimination did not predict ASD-related behaviors measured by the ADOS (R^2^ = 0.005, t = − 0.92, p = .36), CBCL **(**R^2^ = 0.005, t = 0.92, p = .36), or M-CHAT (R^2^ = 0.002, t = − 0.64, p = .61; Table 5). SES also did not predict ASD-related behaviors measured by the ADOS (R^2^ = 0.004, t = − 0.87, p = .38), CBCL **(**R^2^ = 0.01, t= − 1.44, p = .15), or M-CHAT (R^2^ = 0.003, t = − 0.71, p = .48; Table 6).

**Table 4:** Linear regression models examining stress as a main effect and moderator

	ADOS			M-CHAT			CBCL		
	Standardized β	t	Sig	Standardized β	t	Sig	Standardized β	t	Sig
*Block 1: Covariates*									
Biological sex	− .24	− 3.21	.002**	− .10	− 1.26	.21	− .10	− 1.2	.22
Prenatal tobacco use	.15	1.92	.06	.08	.99	.33	.10	1.19	.24
Prenatal alcohol use	− .01	− .10	.92	− .01	− .10	.92	− .15	− 1.81	.07
Prenatal other drug use	.13	1.67	.10	.06	.72	.47	.11	1.35	.18
*Block 2: Main effects*									
Prenatal cannabis use	− .08	− .97	.33	− .12	− 1.41	.16	.13	1.71	.09
Stress	− .03	− .35	.73	.07	.86	.39	.27	3.72	< .001**
Block 3: Interaction*									
Prenatal cannabis use × Stress		− .08	.94		− 1.26	.21		− .04	.97

*SPSS PROCESS does not generate standardized β values

**Correlation is significant at the 0.01 level (2-tailed)

**Table 5:** Linear regression models examining racial discrimination as a main effect and moderator

	ADOS			M-CHAT			CBCL		
	Standardized β	t	Sig	Standardized β	t	Sig	Standardized β	t	Sig
*Block 1: Covariates*									
Biological sex	− .24	− 3.21	.002**	− .10	− 1.26	.21	− .10	− 1.24	.22
Prenatal tobacco use	.15	1.92	.06	.08	1.00	.33	.10	1.19	.24
Prenatal alcohol use	− .01	− .10	.92	− .01	− .10	.92	− .15	− 1.81	.07
Prenatal other drug use	.13	1.66	.10	.06	.72	.47	.11	1.35	.18
*Block 2: Main effects*									
Prenatal cannabis use	− .08	− 1.00	.33	− .12	− 1.45	.15	.13	1.57	.12
Discrimination	− .07	− .94	.35	− .05	− .667	.50	.08	.96	.34
Block 3:Interaction*									
Prenatal cannabis use × Discrimination		.06	.95		.50	.62		− 1.04	.30

*Correlation is significant at the 0.05 level (2-tailed)

**Correlation is significant at the 0.01 level (2-tailed)

**Table 6:** Linear regression models examining SES as a main effect and moderator

	ADOS			M-CHAT			CBCL		
	Standardized β	t	Sig	Standardized β	t	Sig	Standardized β	t	Sig
*Block 1: Covariates*									
Biological sex	− .24	− 1.92	.002**	− .10	− 1.26	.21	− .10	− 1.24	.22
Prenatal tobacco use	.15	1.92	.06	.08	.99	.33	.10	1.19	.24
Prenatal alcohol use	− .01	− .10	.92	− .01	− .10	.92	− .15	− 1.81	.07
Prenatal other drug use	.13	1.66	.10	.06	.72	.47	.11	1.35	.18
*Block 2: Main effects*									
Prenatal cannabis use	− .10	− 1.22	.22	− .14	− 1.67	.10	.10	1.22	.22
SES	− .09	− 1.15	.25	− .09	− 1.11	.27	− .09	− 1.09	.28
Block 3: Interaction*									
Prenatal cannabis use × SES		.45	.65		− .99	.32		− 1.11	.27

*SPSS PROCESS does not generate standardized β values

**Correlation is significant at the 0.01 level (2-tailed)

#### Maternal Stress as a Moderator in Prenatal Cannabis-ASD Association

To test our third hypothesis, we examined whether interactions between (a) prenatal cannabis use and prenatal maternal distress, (b) prenatal cannabis use and racial discrimination, and (c) prenatal cannabis use and SES significantly predicted ASD-related behaviors above and beyond main effects. Despite finding no associations between prenatal cannabis use and ASD-related behaviors, we proceeded with this third aim because moderating variables at times mask a main effect. We found that SES, experiences of discrimination, and maternal stress did not interact with cannabis to predict any of the ASD measures when sex and other prenatal drug exposures were included in the model (see the last row of Tables 4, 5 and 6).

#### Post-Hoc Analyses

To further assess whether our data offers evidence in favor of a nonassociation, we used Bayesian information criteria (BIC) to calculate Bayes factors (see Dienes, 2014). The Bayes factor B compares an alternative hypothesis to the null hypothesis means that the data are B times more likely under the alternative than under the null. We included the same covariates in these analyses as we did for the analyses described above. An estimated Bayes factor indicated evidence in favor of a nonassociation between prenatal cannabis use and ASD behaviors measured by the M-CHAT (B = 1.27), CBCL (B = 0.71), and ADOS (B = 0.67). The estimated Bayes factors for Aim 2 provided strong evidence for the effect of prenatal stress on the CBCL (B = 93.79), but no evidence for the effect of prenatal stress on the M-CHAT (B = 0.56), or the ADOS (B = 0.37).

We also further examined the positive correlation found between prenatal tobacco use and ASD-related behaviors as measured by the ADOS. Using ANCOVA, we found that self-reported tobacco use during pregnancy predicted more ASD-related behaviors observed in the ADOS (F_(1,170)_ = 4.92, p = .03). This finding held when sex, SES, prenatal cannabis use, prenatal alcohol use, and other illicit substance use were included in the model as covariates ADOS (F_(1,165)_ = 4.35, p = .04).

Finally, we completed post-hoc analyses including preterm birth as a predictor in all models. In our sample, preterm birth was positively associated with prenatal cannabis use. We did not include it as a covariate in our primary analyses as we conceptualized it as a potential mediator, rather than a confound, in the relationship between prenatal exposures and ASD-related behaviors. Post-hoc analyses including preterm birth as a predictor were consistent with our primary findings, and did not support the role of preterm birth as a mediator in our sample.

## Discussion

This study examined the association between cannabis use in pregnancy and child ASD-related behaviors and evaluated several components of maternal stress as moderators of this relationship. We found no significant associations between prenatal cannabis use and ASD-related behaviors. These findings add to the mixed literature on cannabis-ASD associations. Although some recent reports have indicated an association between higher levels of prenatal cannabis exposure and ASD (Corsi et al., 2020; Wood et al., 2015), others have not (DiGuiseppi et al., 2021). However, these studies used diagnostic categories of ASD and retrospective maternal self-report which is subject to recall-bias (Reece & Hulse, 2020). By utilizing continuous measures of ASD-related behaviors, we were better able to capture the extent to which prenatal cannabis use and stress impact the full spectrum of these behaviors. Additionally, the current study was prospective: mothers were asked about their substance use during their pregnancy by clinical providers and research staff.

Our sample consisted of Black mother child dyads, who have not been well represented in previous literatures and for whom disparities in ASD care have been noted. To our knowledge, only one other study has examined ASD behaviors in a sample comprised entirely of Black mothers (Constantino et al., 2020). This within-group evaluation is especially important given the aforementioned racial disparities in maternal prenatal stress and ASD prevalence. This work will contribute to a better understanding of within-race risk for ASD, providing insight into risk and protective factors relevant to Black American families.

Furthermore, the majority of the studies in the stress and ASD literature also evaluate only a single component of maternal stress. Turner and Avison (2003) compared a life events checklist alone to a wider range of measures of stress–recent life events, chronic stressors, lifetime major events, and discrimination and found that life events alone systematically underestimated stress exposure among Black Americans relative to their White counterparts and among persons of lower SES relative to their more advantaged counterparts. Therefore, by employing similarly comprehensive methods of evaluating stress, and examining the distinct impacts of experiences of discrimination, the current study better addresses the relevant stressors in Black American communities.

Our findings suggest that other prenatal risks might be more pertinent than prenatal cannabis use in the prediction of child ASD-related behaviors, namely prenatal maternal distress and prenatal tobacco use. These results replicate previous findings in the literature (Hertz-Picciotto et al., 2022; Kinney et al., 2008), which validates the measurement and sensitivity of ASD behaviors in our sample. Furthermore, this study extends findings to a Black American sample.

It is possible that both prenatal distress and tobacco exposure cause epigenetic changes relevant to ASD risk. For example, when examining DNA methylation in infants, Hannon et al. (2018) identified epigenetic signatures of gestational age and prenatal tobacco exposure, although specific genetic loci associated with ASD were not identified in this sample. Additionally, the genetic underpinnings of serotonin and dopamine signaling and stress physiology mechanisms have been shown to be particularly relevant to social, attentional, and internalizing behavioral changes (Abbott et al., 2018).

Contrary to our expectations, children were not at greater risk for ASD-related outcomes if their mothers used cannabis and were exposed to high levels of stress. It might be that other combinations of other types of prenatal exposures are more relevant to ASD risk, such as air pollutants or chemical toxicants in combination with prenatal stress. Future studies should explore this possibility, particularly in Black samples where structural racism might lead to greater exposures to potentially harmful toxicants. For example, Black people and individuals of low SES are more likely to be exposed to air pollution due to residential segregation (Alvarez, 2022; Brailsford et al., 2018; Bravo et al., 2016).

### Strengths and Limitations

Strengths of the current study included the use of a prospective research design and continuous measures of ASD-related behaviors and the inclusion of multiple measures of each construct. In addition, we examined potential confounds including prenatal exposure to tobacco and child sex, both of which have been found to significantly predict ASD outcomes. Another strength of the current study is the high rate of cannabis users in our sample. Clinical reports of cannabis use prevalence during pregnancy vary widely from 3% to upwards of 35% in North America (Nashed et al., 2021). Our sample reported cannabis use during pregnancy at the upper end of this range with 35% of mothers self-reporting cannabis use. This high rate of use would allow us to detect most effects, save a small effect size, which increases our confidence that such an association truly does not exist. Results of our Bayesian models further supported the results and conclusions from our frequentist analyses.

Another strength of the current study is that it evaluated associations in an all Black sample. When racial disparities are reported, it is often assumed that this is due to experiences of discrimination and/or SES, but we were able to specifically examine these potential confounds. It may, however, be the case that Black Americans of lower SES were overrepresented in our sample. 45% of our sample reported an income below the poverty level, whereas a recent census data estimated a poverty rate of 19% among Black Americans (Creamer, 2020).

This study also utilized a community sample with relatively low rates of ASD symptoms. It is possible that the children in our sample may have had lower rates of ASD behaviors due to attrition, as parents of children with more severe behavioral presentations may have been less likely to attend the follow-up visit. If this were the case, our findings may be biased towards the null as we would not be accounting for more severe presentations of ASD. Although ASD is reliably diagnosed at age 2, research shows that Black children tend to be diagnosed later than White children, likely due to limited access to care and provider biases (Maenner et al., 2020; Mandell et al., 2002). A longitudinal study that extends to later child ages to assess ASD-related behaviors may yield different results.

The current study examined the role of child biological sex, one of the best known risk factors for ASD, in the association between prenatal cannabis exposure and ASD-related behaviors. ASD is 4.3 times as prevalent among boys than girls (Maenner et al., 2020). Prenatal cannabis use also appears to affect males differently than females. Using an animal model, Frau et al. (2019) found that male rats that were exposed to THC *in utero* exhibited a hyperdopaminergic state that led to increased behavioral sensitivity in preadolescence similar to the sensitivities characteristic of ASD. In contrast, prenatal THC does not affect the mesolimbic dopaminergic system, or socioemotional behavior, in preadolescent female rats (Traccis et al., 2020). Therefore, it is possible that sex moderates the association between prenatal cannabis use and ASD behaviors, a possibility that was not examined in the current study.

Because we utilized a PCA approach to some of our main variables of interest, we cannot rule out the possibility of residual confounding. PCAs were completed to reduce the number of variables that might represent maternal stress or stressors, in order to reduce type I error when testing hypotheses.

Because we used medical record review data to measure maternal cannabis use during pregnancy, we did not have data on the timing of the cannabis exposures. The timing of other exposures such as prenatal stress, trauma, nutrition, ethanol, and alcohol, has been associated with neurodevelopmental outcomes (Davis & Sandman, 2010; Mooney & Varlinskaya, 2011; O’Leary et al., 2010; Zhang et al., 2018). Future work should examine if such a pattern exists for prenatal windows of exposure to cannabis.

Additionally, this study utilized self-reported substance use which is vulnerable to underreporting due to social desirability—some mothers might feel shame, be fearful of being judged, and/or fearful of facing legal repercussions for reporting substance use (Vergés, 2022). This may have been especially true for our sample of Black women given that Black newborns were four times more likely than White newborns to be reported to Child Protective Services at delivery based on alcohol/drug use identified by prenatal care providers, despite having at similar rates to White women (Roberts & Nuru-Jeter, 2012). It is also possible that social desirability biases vary by levels of depressive symptoms based on Latkin et al’s (2017) research with opiate and cocaine users. Social desirability could have led participants to report less substance use than had actually occurred. To help diminish this effect, future work should measure cannabis exposure using biological assays to quantify THC levels. In this way, a dose-response effect could also be examined. In this study, THC data were only available for a subsample of 77 participants, prohibiting their use in primary analyses (due to statistical power concerns). We did find that higher concentrations of THCA were significantly associated with positive self-reports of cannabis use in that subsample.

Relatedly, most of the research examining cannabis biomarkers solely focuses on THC, and not the other components of cannabis including cannabinoidol (CBD), which could alter the how the compounds are processed. In fact, some research suggests that CBD may have a protective cognitive effect when consumed in tandem with THC (Madras, 2019). This is especially relevant as there are currently no government regulations on THC:CBD ratios (Zeyl et al., 2020). It is also possible that the method of consumption (i.e. smoking versus vaping versus eating) could alter how it’s metabolized and therefore how it affects the fetus. Moreover, little research has examined the impact of postnatal cannabis exposure including transfer through breastfeeding. Future work is needed to better assess these unaddressed issues.

## Conclusion

In conclusion, we replicated previous findings that higher levels of maternal stress are associated with higher levels of reported ASD behaviors in two-year-old children. We found no evidence that prenatal cannabis exposure increases risk for ASD-related behaviors in a sample of Black children. It is still possible, however, that prenatal cannabis exposure can affect other facets of child development and behavior that were outside of the scope of this study. Findings from this study could inform policies and recommended guidelines regarding maternal cannabis use in pregnancy, which has become more commonplace in response to changing laws and the medicinal properties of this drug.

## Supplementary Information

Below is the link to the electronic supplementary material.

Supplementary file1 (PDF 51 kb)

Supplementary file2 (PDF 51 kb)

Supplementary file3 (PDF 93 kb)

Supplementary file4 (PDF 24 kb)
