# Supplementary material for: Prenatal Cannabis Use and Offspring Autism-Related Behaviors: Examining Maternal Stress as a Moderator in a Black American Cohort
Source: J Autism Dev Disord. 2023 Apr 25;54(6):2355–67. doi: 10.1007/s10803-023-05982-z (PMC10127191; doi:10.1007/s10803-023-05982-z)
Supplement: Supplementary file 1 — Supplementary file1 (PDF 51 kb) [file 10803_2023_5982_MOESM1_ESM.pdf]

Principal Components Analysis: N=172 participants in current study

Total Variance Explained

Component	Initial Eigenvalues			Extraction Sums of Squared Loadings			Rotation Sums of Squared Loadings[a]
	Total	% of Variance	Cumulative %	Total	% of Variance	Cumulative %	Total
1	4.556	35.044	35.044	4.556	35.044	35.044	4.385
2	2.604	20.028	55.072	2.604	20.028	55.072	2.571
3	1.404	10.804	65.876	1.404	10.804	65.876	2.575
4	.799	6.144	72.020				
5	.674	5.182	77.202				
6	.591	4.547	81.748				
7	.538	4.136	85.884				
8	.496	3.812	89.696				
9	.360	2.768	92.465				
10	.344	2.649	95.114				
11	.274	2.111	97.225				
12	.223	1.714	98.940				
13	.138	1.060	100.000				

Extraction Method: Principal Component Analysis.

a. When components are correlated, sums of squared loadings cannot be added to obtain a total variance.

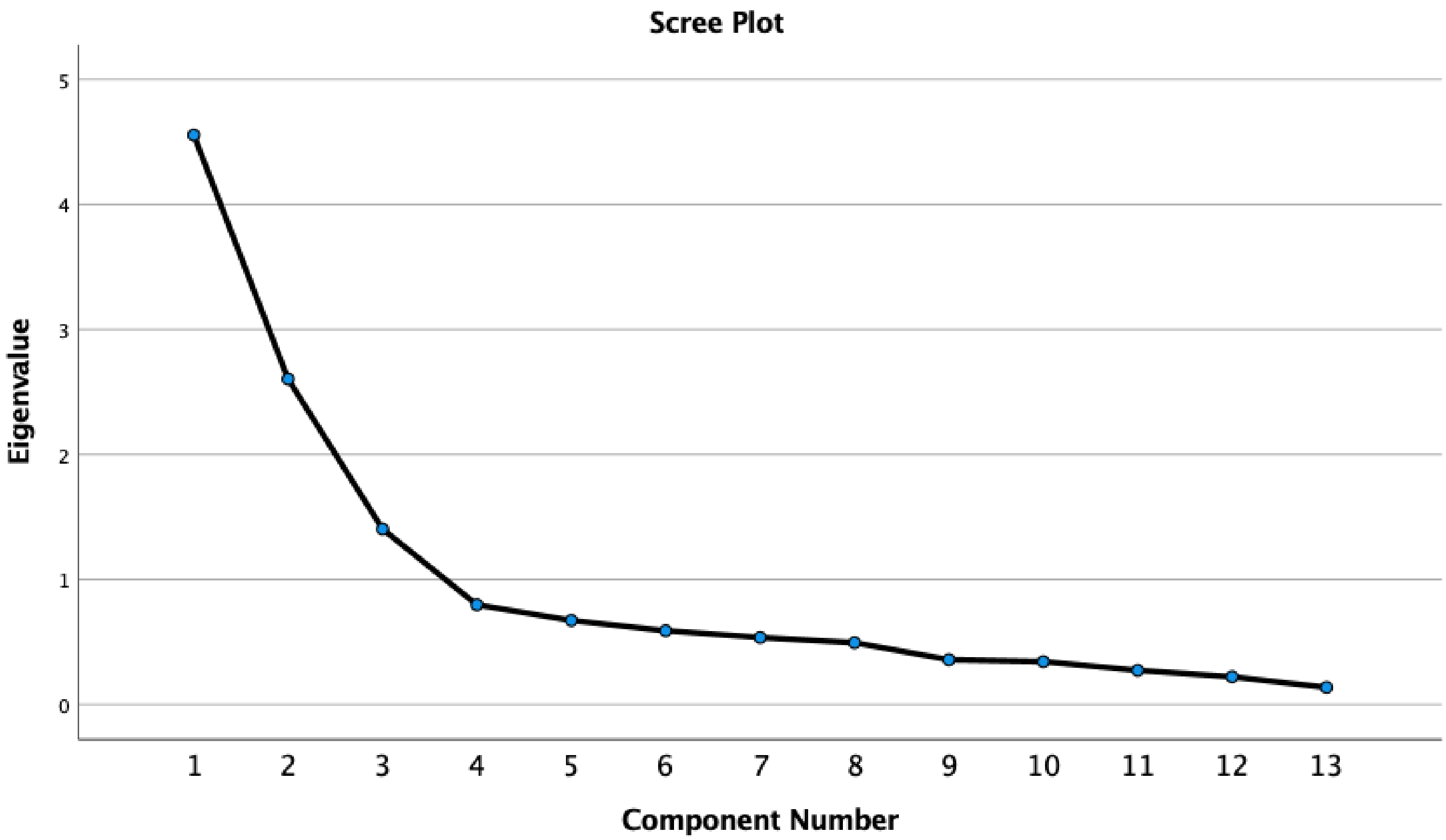
Scree Plot
Eigenvalue
Component Number
0
1
2
3
4
5
1
2
3
4
5
6
7
8
9
10
11
12
13

Pattern Matrix[a]

	Component		
	1	2	3
Marital Status		.627	
Education		.699	
Income		.868	
Insurance		.871	
PSS Visit 1	.810		
Edinburgh Visit 1	.756		
STAI Visit 1	.828		
PSS Visit 2	.869		
Edinburgh Visit 2	.793		
STAI Visit 2	.853		
ACES			.898
CTQ			.746
Stressful Life Events Inventory			.671

Extraction Method: Principal Component Analysis.
Rotation Method: Promax with Kaiser Normalization.[a]
a. Rotation converged in 4 iterations.
