# Supplementary material for: Prenatal Cannabis Use and Offspring Autism-Related Behaviors: Examining Maternal Stress as a Moderator in a Black American Cohort
Source: J Autism Dev Disord. 2023 Apr 25;54(6):2355–67. doi: 10.1007/s10803-023-05982-z (PMC10127191; doi:10.1007/s10803-023-05982-z)
Supplement: Supplementary file 2 — Supplementary file2 (PDF 51 kb) [file 10803_2023_5982_MOESM2_ESM.pdf]

Principal Components Analysis: All Prenatal Participants

Total Variance Explained

Component	Initial Eigenvalues			Extraction Sums of Squared Loadings			Rotation Sums of Squared Loadings[a]
	Total	% of Variance	Cumulative %	Total	% of Variance	Cumulative %	Total
1	4.510	34.691	34.691	4.510	34.691	34.691	4.315
2	2.424	18.643	53.334	2.424	18.643	53.334	2.510
3	1.337	10.287	63.621	1.337	10.287	63.621	2.548
4	.755	5.810	69.432				
5	.720	5.542	74.973				
6	.654	5.033	80.006				
7	.564	4.341	84.347				
8	.489	3.760	88.107				
9	.419	3.226	91.333				
10	.370	2.848	94.181				
11	.349	2.687	96.868				
12	.224	1.722	98.590				
13	.183	1.410	100.000				

Extraction Method: Principal Component Analysis.

a. When components are correlated, sums of squared loadings cannot be added to obtain a total variance.

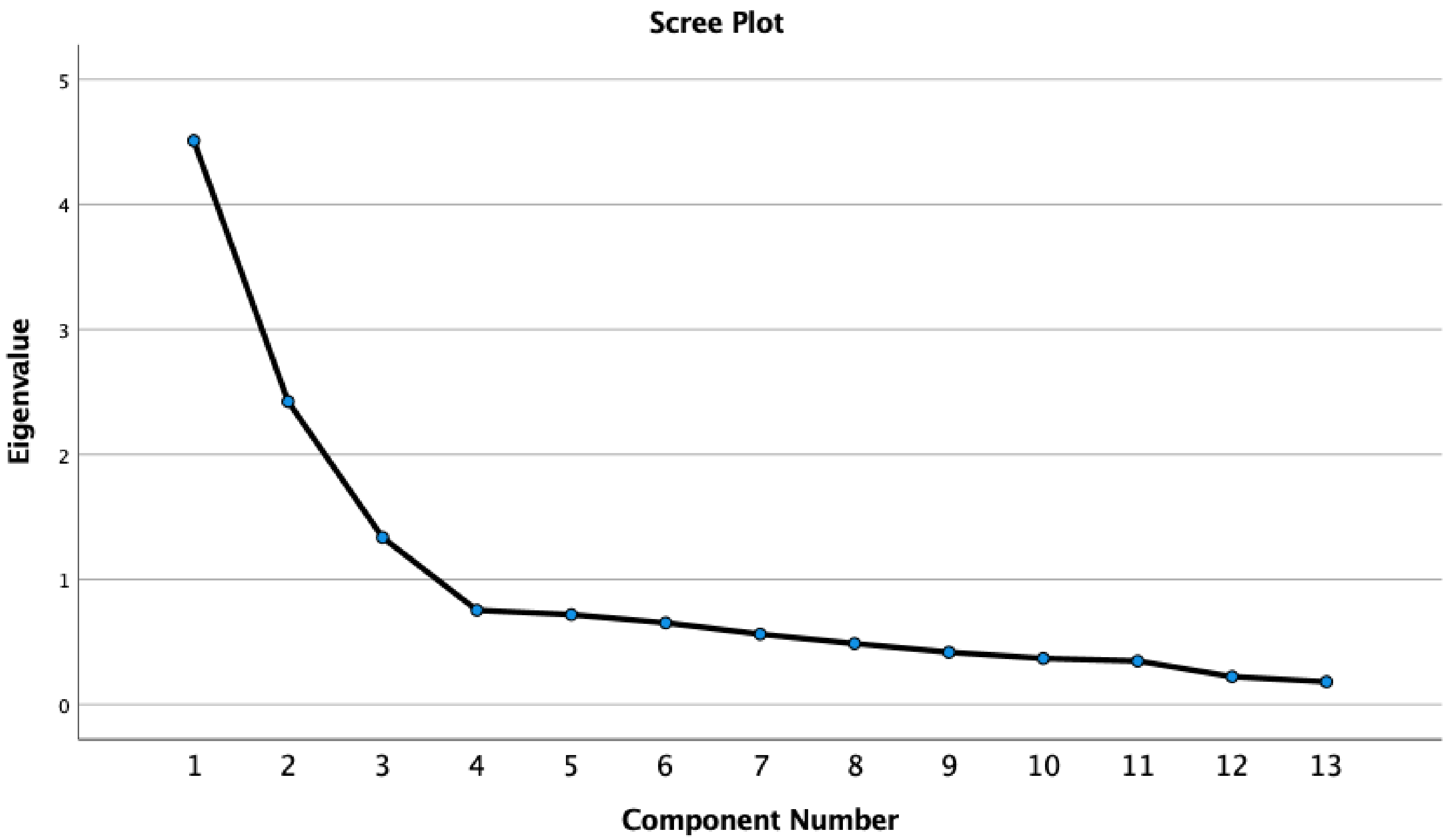
Scree Plot
Eigenvalue
5
4
3
2
1
0
1
2
3
4
5
6
7
8
9
10
11
12
13
Component Number

Pattern Matrix[a]

	Component		
	1	2	3
Marital Status		.641	
Education		.746	
Income		.853	
Insurance		.813	
PSS Visit 1	.770		
Edinburgh Visit 1	.757		
STAI Visit 1	.815		
PSS Visit 2	.852		
Edinburgh Visit 2	.774		
STAI Visit 2	.856		
ACES			.895
CTQ			.596
Stressful Life Events Inventory			.810

Extraction Method: Principal Component Analysis.
Rotation Method: Promax with Kaiser Normalization.[a]
a. Rotation converged in 4 iterations.
