# Supplementary figures and images for: Prenatal Cannabis Use and Offspring Autism-Related Behaviors: Examining Maternal Stress as a Moderator in a Black American Cohort
Source: J Autism Dev Disord. 2023 Apr 25;54(6):2355–67. doi: 10.1007/s10803-023-05982-z (PMC10127191; doi:10.1007/s10803-023-05982-z)

# Conceptual Model

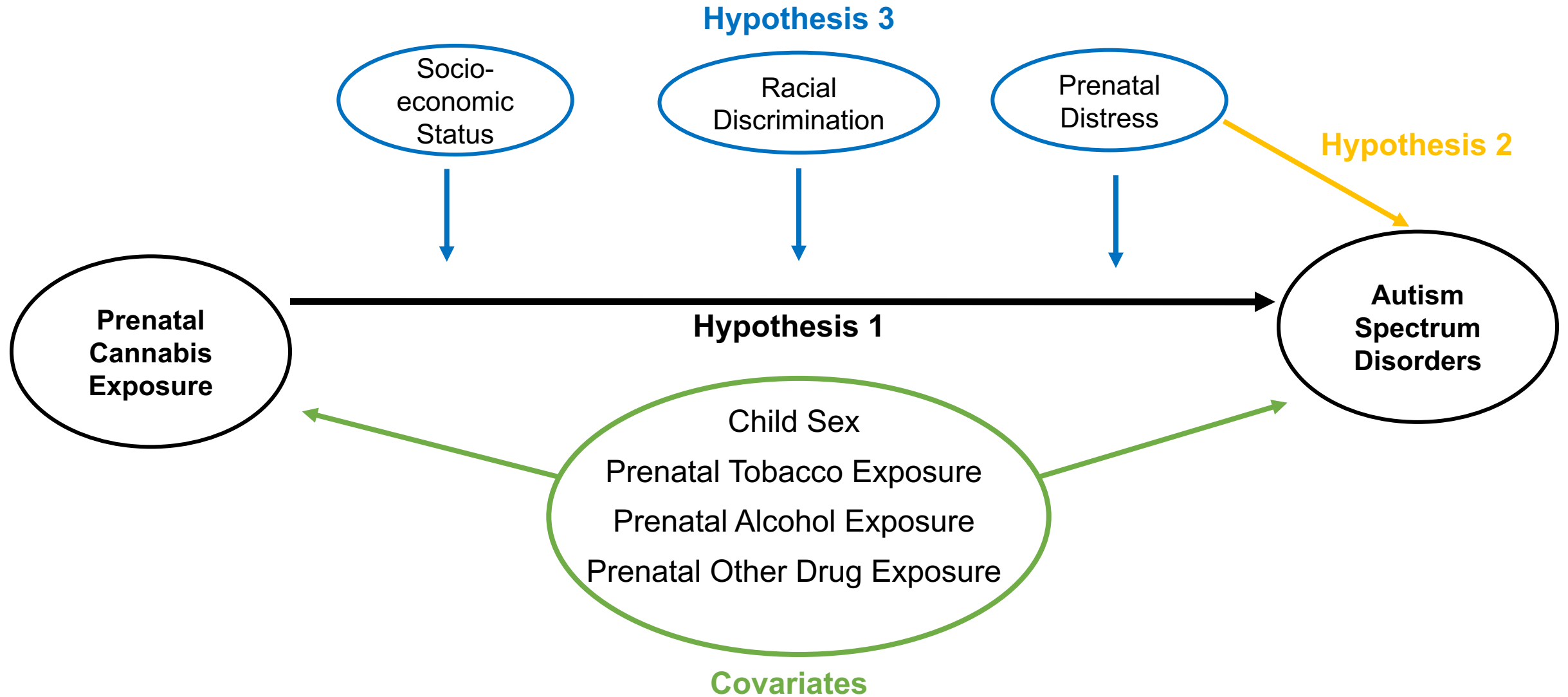

Supplement: Supplementary file 3 — Supplementary file3 (PDF 93 kb) [file 10803_2023_5982_MOESM3_ESM.pdf]
