# Supplementary material for: Prenatal Cannabis Use and Offspring Autism-Related Behaviors: Examining Maternal Stress as a Moderator in a Black American Cohort
Source: J Autism Dev Disord. 2023 Apr 25;54(6):2355–67. doi: 10.1007/s10803-023-05982-z (PMC10127191; doi:10.1007/s10803-023-05982-z)
Supplement: Supplementary file 4 — Supplementary file4 (PDF 24 kb) [file 10803_2023_5982_MOESM4_ESM.pdf]

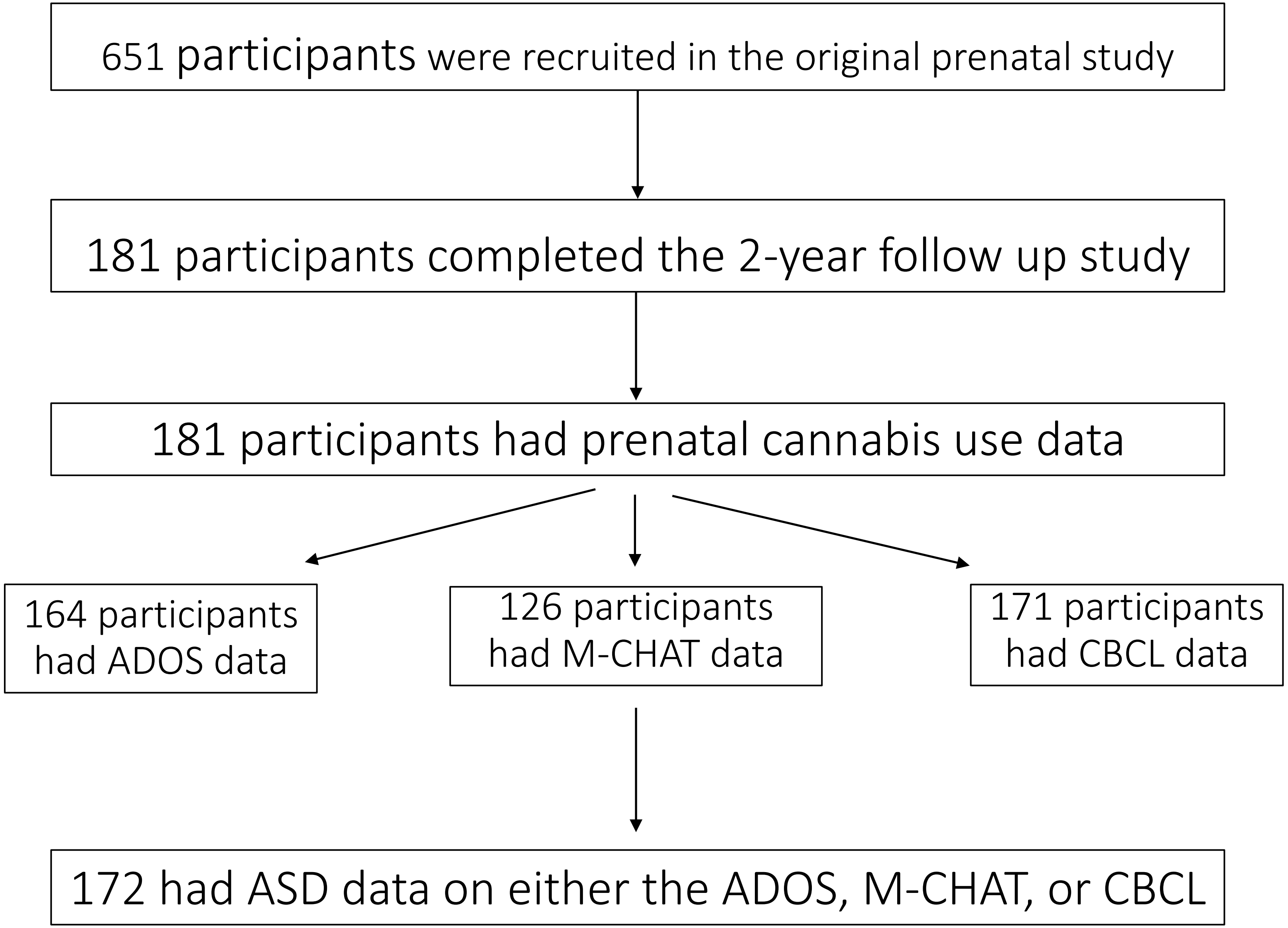

651 participants were recruited in the original prenatal study
181 participants completed the 2-year follow up study
181 participants had prenatal cannabis use data
164 participants had ADOS data
126 participants had M-CHAT data
171 participants had CBCL data
172 had ASD data on either the ADOS, M-CHAT, or CBCL
